# International Prolonged Grief Disorder Scale Addendum for Refugees and Displaced People (IPGDS-ARD): A Study of Arabic-Speaking Bereaved Refugees

**DOI:** 10.32872/cpe.11435

**Published:** 2025-02-28

**Authors:** Clare Killikelly, Alexandra Reymond, Anaïs Aeschlimann, Andreas Maercker, Eva Heim

**Affiliations:** 1 Department of Psychology, University of Zurich, Zurich, Switzerland; 2 Department of Psychiatry, University of British Columbia, Vancouver, Canada; 3 Institute of Psychology, University of Lausanne, Lausanne, Switzerland; Friedrich-Alexander-Universität Erlangen-Nürnberg, Erlangen, Germany

**Keywords:** grief, bereavement, prolonged grief disorder, cross-cultural relevance

## Abstract

**Background:**

Prolonged grief disorder (PGD) is a new and significant addition to the ICD-11 WHO disease classification system and the DSM 5-TR. As a new disorder, it stands to improve diagnostic precision, enhance communication among health professionals and patients, provide better access to care and lead to effective treatments and intervention. However, it remains to be determined if the new diagnostic criteria for PGD are applicable to different cultural groups.

**Method:**

Here we sought to adapt the International Prolonged Grief Disorder Scale for refugees and displaced people. We conducted two focus groups with clinicians and health care workers and six cognitive interviews with bereaved Arabic-speaking refugees.

**Results:**

This formative research resulted in an addendum (comprised of three new scales) to the IPGDS aimed to aid with treatment planning: the 42 item Addendum for Refugees and Displaced people (IPGDS-ARD). Here we present the steps for scale augmentation based on cultural considerations, a detailed description of clinical utility, feasibility and content validity established at each step, and an analysis of the percent of change in content at each step.

**Conclusion:**

We conclude that the presented method of scale augmentation is a feasible and efficient approach that led to a culturally relevant, clinically useful addendum to an existing PGD questionnaire.

## Highlights

- This study is one of the first formative research studies on culturally relevant items for treatment planning with bereaved refugees.
- The additional scales developed seek to unpick the complex experiences of ambiguous loss, adjustment to host country and grief.
- With this work we present a detailed description of qualitative research methods for scale augmentation for bereaved refugees.

Researchers and clinicians are increasingly confronted with a difficult and exciting question: to what extent does cultural background contribute to the presentation, chronicity, and treatment of mental health disorders? The WHO’s ICD-11 and the DSM-TR 5 now include cultural caveats in their definitions of mental disorders (Boelen et al., 2020; Killikelly & Maercker, 2017; Prigerson et al., 2021). For example, the new diagnostic definition of prolonged grief disorder (PGD) can only be diagnosed if symptoms persist for a longer period of time or are more intense than would be expected in the individuals’ culture and context (Maercker et al., 2013). This novel addition to diagnostics catalyzes important discussions about the role of culture in mental disorder presentation and treatment. However, there are several challenges that have so far been overlooked. It is not clear how to establish the cultural norms of an individual or culture and whether these have been violated by disorder. For example, in the case of grief, cultural norms prescribe a mourning period and if grieving persists beyond the culturally expected mourning period that cultural norm has been violated. Clinicians, researchers, and health care workers on the frontline of mental health assessment are perplexed by the proposition that culture can quickly and easily be assessed in a diagnostic setting (Stelzer et al., 2020). The new disorder definition of PGD provides a unique opportunity to explore the assessment of a mental health disorder with its emphasis on culture and global applicability (First et al., 2015). Core symptoms of PGD include longing for and preoccupation with the deceased, significant emotional distress and significant functional impairment that persist beyond half a year after the death of a loved one (Killikelly & Maercker, 2017). Symptoms can differ in duration and expression according to the culture, religion, social status or gender of the bereaved (Rosenblatt, 2008). Therefore, although six months seem to be a good approximation of a grieving process, this duration is not exclusively limited. Indeed, the main distinction in diagnostic criteria between the newly proposed ICD-11 and DSM 5-TR PGD is the time criteria: the ICD-11 purports that symptoms must persist for more than six months, and the DSM suggests more than 12 months. To date, when questions about the duration of symptoms arise, clinicians are advised to use cultural norms in diagnostic decision making (O’Connor et al., 2015). Currently there is only one PGD assessment questionnaire that includes an item examining the role of culture. The International ICD-11 Prolonged Grief Disorder Scale (IPGDS) was developed based on key ICD-11 items from the Prolonged Grief Disorder-13 (PG-13; (Prigerson & Maciejewski, 2008) and the Structured Clinical Interview for Complicated Grief (SCI-CG; Bui et al., 2015; Killikelly et al., 2020). This questionnaire was developed in two parts. Part one examines the specific diagnostic items of the ICD-11 (13 items) and includes one item related to the cultural caveat (Item 14 *My grief would be considered worse [e.g., more intense, severe and/or of longer duration] than for others from my community or culture)*. Part one of this scale may be used to establish a preliminary diagnosis of PGD according to established diagnostic features. Part two of the scale is a catalogue of possible grief symptoms that may have a stronger cultural fit depending on the individual assessed, (for example Item 4: I had a strong loud emotional outburst after the loss) (Killikelly & Maercker, 2023). Part two provides the patient and clinician a more in-depth assessment tool that may help with treatment planning, therapeutic rapport and further symptom delineation but is not used for diagnostics.

This two-part assessment framework is modeled on the Harvard Trauma Questionnaire (Mollica et al., 1992). The HTQ was developed in several parts and contains a combination of checklist and open-ended questions about traumatic events and emotional symptoms that are unique to a particular place and context. The HTQ is intended to be used in clinician interviews and not as a self-report. We have built on this framework by including a standard scale of well-known PGD symptoms alongside a supplementary assessment of additional symptoms that may be culturally relevant and particularly relevant for treatment planning. In addition, we provide a new simplified framework for how to adapt the IPGDS to different cultural groups. Presently, the IPGDS has been used and adapted to assess PGD and wider symptoms of grief in German-speaking, Chinese, Japanese, Syrian bereaved and a group of Arabic speaking migrants (Killikelly & Maercker, 2023). The standard scale and the adapted cultural supplement have been psychometrically validated in German-speaking and Chinese bereaved and preliminary validated in a group of Swiss and Canadian migrants from a variety of backgrounds (Killikelly et al., 2020). The cultural supplement was found to be valid and reliable for use in each of these groups and in each case items were augmented with specific culturally relevant items. For example, Chinese participants requested and strongly endorsed Item 1 of the cultural supplement ‘I experience strong physical problems since the loss (e.g., headache, problems with appetite)’.

Humanitarian migrants comprise refugees, asylum seekers or displaced people with a population rising to more than 70 million around the world (UNHCR, 2017). This group is affected by several challenges that have a negative impact on mental health including a high rate of traumatic events (Bhugra & Becker, 1999). The most common disorders are posttraumatic stress disorder (PTSD), depression and anxiety disorder (Fazel et al., 2005). Concerning PGD, Killikelly et al. (2018) found that refugees are significantly more affected than the normal population. For example up to 54% of refugees may experience symptoms of PGD while less than 10% of the general population (Lechner-Meichsner et al., 2024; Rosner et al., 2021). Research has shown that ethnic minorities, refugees and immigrants are more likely to be misdiagnosed with mental health disorders than patients from the main culture (Bäärnhielm et al., 2015). Indeed, patients and clinicians’ differences such as culture, gender, language, religion and ages can lead to a misunderstanding of the illness (Lewis-Fernández & Kirmayer, 2019). Currently there is an urgent need for a measure of PGD that is relevant and acceptable for refugees and displaced people. The main aim of this study is to culturally refine and develop a PGD addendum for refugees and displaced people that considers the distinctive experiences of refugees. This addendum may be used to supplement to the standard ICD-11 PGD diagnostic items (IPGDS standard scale) in the effort to improve therapeutic rapport, identify missing symptoms or features and improve treatment and care planning with refugees. As a proof of concept, we have explored symptoms and experiences of grief in a small group of Arabic-speaking refugees.

## Augmentation of the IPGDS for Arabic-Speaking Refugees

**Figure 1:**
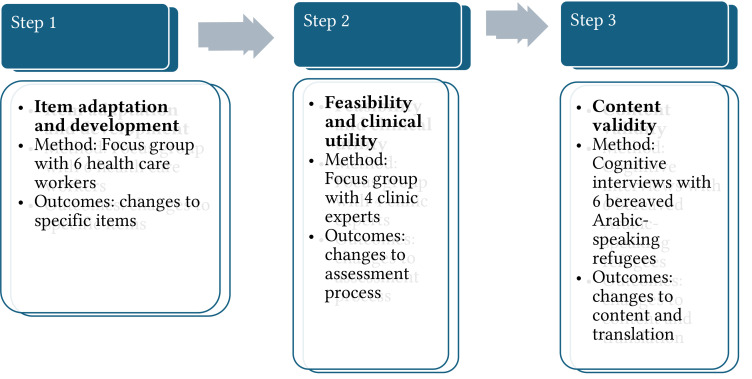
Iterative Process of Cultural Augmentation and Testing

According to Boateng et al. (2018) there are three phases to scale augmentation (item development, scale development, scale evaluation) including nine steps to create and validate a scale. This project has developed a simplified method that focuses on the two steps of the “item development” phase (identification of domain and item generation, content validity), and on the first step of the “scale development” phase, which consists in the pre-testing of questions to distill the most relevant and important information for cultural relevance (see Figure 1).

Two focus groups (FGs) were conducted: the first focused on item generation and adapting the content of the questionnaire and a second focused on determining the feasibility and clinical utility of the questionnaire. The following research questions were addressed in the focus groups:

1. What content (specific items) from the existing IPGD scale is missing / needs to be added for bereaved Arabic-speaking refugees?
2. Is the IPGDS feasible for use with Arabic-speaking refugees in different clinical settings? Is the IPGDS for refugees clinically useful?

The World Health Organization (WHO) Procedure of translation and adaptation of instruments was followed to translate the IPGDS for refugees in Arabic (World Health Organization, 2019). To establish the preliminary content validity of the questionnaire in Arabic, six cognitive interviews (CIs) were conducted with bereaved Arabic-speaking refugees (5 Syrian, 1 Iraqi) to evaluate the possible sources of errors in the questionnaire and acceptability of the items (Beatty & Willis, 2007). The following research question was examined in the CI’s:

- Is the content of the IPGDS for Arabic-speaking refugees valid and acceptable?

## Method

### Procedure

Ethical approval was obtained from the UZH Faculty of Arts and Social Sciences (Grant No. 19.10.4.)

#### Measure: IPGDS

This scale comprises 13 previously used items integrating the PG-13 (Prigerson & Maciejewski, 2008) and the SCI-CG (Bui et al., 2015). The participants indicated how often they felt preoccupation, yearning and symptoms of emotional distress over the past month because of loss of a loved one, using a 5-point scale: 1 = almost never (less than once a month), 2 = rarely (monthly), 3 = sometimes (weekly), 4 = often (daily), and 5 = always (several times a day). An impairment item as well as screening items (for the length of time since bereavement and the violation of socio-cultural norms) were also included. As mentioned above Item 14 assesses the cultural caveat. The original version of the cultural supplement consisted of 19 items developed from key informant interviews with health care workers and bereaved individuals from Europe and China (Stelzer et al., 2020). Psychometric analysis confirmed the internal consistency, concurrent and criterion validity. In this current study we aim to develop a new cultural supplement, now referred to as the addendum, from focus groups and interviews with bereaved refugees and migrants.

#### Step 1: Item Generation and Augmentation of IPGDS for Refugees Using a Focus Group

The first FG was organized to generate new possible questionnaire items and discuss the adaptation of the IPGDS for Arabic-speaking refugees. Five professional health care workers were invited to discuss the questions of the IPGDS for refugees in general and evaluate items’ relevance for the bereaved Arabic-speaking refugees in particular. This first meeting took place in December 2019 at the University of Zürich. The meeting lasted 2 hours and was facilitated by CK, Post Doctoral Researcher and Clinical psychologist with support from Master’s students AR and AA. The purpose of the meeting was explained and the experts were asked to share their personal experiences of working clinically with bereaved refugees, to assess what should be added or could be missing from the standard IPGDS questionnaire, and to share their feedback/comments. The meeting was audio recorded to collect the data and transcribed with MAXQDA (version 2020). In line with the first step of scale adaptation “identification of domain and item generation”, a draft catalogue of possible grief symptoms was collected based on the FG recommendations and with input from existing literature (Hassan et al., 2016; Kokou-Kpolou et al., 2017; Vromans et al., 2012). These included 57 new possible items were added to the 14 standard items of the International Prolonged Grief Disorder scale (IPGDS) (Killikelly et al., 2020).

#### Step 2: Feasibility and Acceptability of the IPGDS for Refugees Using a Second Focus Group

The second phase of the scale development aimed to assess the feasibility and clinical utility of the questionnaire. For this purpose, a second FG was organized with four other professional health care workers who evaluated the items to determine if the scales would be feasible for use in the clinic setting and if they would be clinically useful. The second FG took place at local psychosomatic clinic at the University Hospital Zurich in December 2019. This focus group lasted one hour. It was facilitated by CK and supported by AR and AA. The purpose of the focus group was explained, participants were asked to provide feedback on the scales, particularly considering the treatment of refugee patients who were suffering from grief.

#### Step 3: Preliminary Content Validity of IPGDS in Refugees Using Cognitive Interviewing

Cognitive interviews (CIs) can be used to clarify how items could be understood or how participants will answer to specific questions (Drennan, 2003). The main goal is to investigate how a participant arrives at an answer instead of the answer itself. There are two different alternatives or paradigms to conduct CIs, the “think-aloud” method and “probing”, which both aim to gather information that can’t be seen in the questionnaire (Beatty & Willis, 2007). In the “think-aloud” method, the interviewer asks the participant to answer the questions by explicitly thinking out loud. It means that the interviewer asks the questions to the participant and looks at how they arrive at their answer. The other method, “verbal probing”, consists of asking the participant the questions and then to elaborate on their answer. Probes can be used to ask about comprehension or interpretation of the questions, to paraphrase the questions, to ask about confidence judgment, to recall things, and to ask about very specific things as well as more general thoughts (Beatty & Willis, 2007). Both “thinking-aloud” and “verbal probing” methods are often used together in CIs as was done in the current study ((Willis & Artino, 2013).

Following the forward and back translation of the questions (WHO procedure), five CIs were conducted to pre-test the IPGDS for refugees. The interviews started with a presentation of the interviewer (AR Master’s student) and the translator. A qualified clinical psychologist was always on hand (CK) in case participants became distressed. Participants were also provided with a list of local resources and psychological services. All five interviews lasted between 60 and 90 minutes. The interviewer explained the purpose of the study and of the interview and gave instructions on how to answer the questions according to the “think-aloud” method. The interviewer used probes to go deeper when insufficient information was given. After five interviews the IPGDS was adapted. A final CI with a sixth person was then conducted to pre-test this new version of the questionnaire. At the end of the interviews, participants received 30 Swiss francs for their participation.

### Recruitment and Participants

#### Focus Group and Cognitive Interviews

The recruitment of professional health care workers for the FGs took place in November and December 2019. Clinicians were mostly recruited from health clinics that specialize in the treatment of migrant and refugee communities, private practices and NGOs in the German-speaking part of Switzerland. The main criteria were “having experience working with refugees/migrants/asylum seekers for at least one year and having professional experience with grief”. The first FG consisted of six health care workers and the second of four clinicians, all of whom specialized in the treatment of trauma and grief in refugee populations.

The recruitment of participants for the CIs started in early January and lasted until the end of February 2020. Participants were first recruited through NGOs, language schools, associations, and refugee housing. The snowballing method was used for further recruitment. Inclusion criteria included speaking Arabic, the ability to provide written informed consent and having lost a loved one at least six months prior to the interview. People with severe mental health disorders (e.g., a diagnosis of major depression or current diagnosis of schizophrenia), imminent risk of suicide, or currently receiving treatment from a psychiatric in-patient unit were excluded from the study. Five participants were from Syria and one from Iraq (Table 1).

**Table 1:** Demographic Information of Cognitive Interview Participants

Category	P1	P2	P3	P4	P5	P6
Gender	Female	Female	Male	Male	Male	Female
Age	28	48	23	48	46	70
Ethnicity	Syrian	Syrian	Syrian/Kurd	Syrian/Kurd	Syrian	Iraq
Residency status	Residency	Asylum Seeker	Waiting to have the refugee status	Temporary Visa	Residency	Asylum Seeker
Cause of migration	Study	War refugee	Political refugee War refugee	Political refugee War refugee	Political refugee	War refugee
Relation to the deceased	Close friend	Parent	Cousin Friends	Parent	Brother	Parent
Cause of the death	Homicide	Natural cause	Homicides	Natural cause	Homicide	Natural cause
Missing person	No	No	No	Yes	No	Yes

### Analyses

The FG and CI data was audio recorded and then transcribed with MAXQDA (version 2020). The framework method of qualitative analysis was used as this method is frequently used for semi-structured interview transcripts (Gale et al., 2013). The framework approach involves the categorization and organization of the qualitative data into a matrix to reduce and summarize the data with the aim to answer the research questions and to generate themes. The framework analysis was conducted according to the seven steps proposed by Gale et al. (2013): “transcription”, “familiarization with the interview”, “coding”, “developing a working analytical framework”, applying the analytical framework”, “charting data into the framework matrix” and “interpreting the data” using Microsoft excel. Regarding the first step of the analysis, all FGs and CIs were transcribed using MAXQDA software. FGs were conducted in English and transcribed into English. CIs were conducted in English with an Arabic speaking translator and transcribed into English. This study received ethical approval from the University of Zurich.

## Results

The results from this study are presented in two formats. The first type of results are the new questionnaire items and the structure for the new measure of grief. The step-by-step questionnaire adaptation and restructuring is presented in Appendix 1 (see Killikelly et al., 2025S). Second, the results from the qualitative/descriptive data analysis of the FG and CI are presented. These results provide an in-depth rationale supporting the amendment and additions to the IPGDS. They are organized into conceptual themes of clinical utility, feasibility, and content validity. The results of the framework analysis for the two FGs (combined) and CIs are presented separately below.

### Focus Groups

The first focus group resulted in a new structure of the IPGDS for Arabic-speaking refugees (see Killikelly et al., 2025S, Appendix 1, Step 1 FG1 outcomes). The cultural supplement was replaced with three new scales a) *Loss of homeland* and b) *Refugee adjustment and impact on grief* and c) *Culturally specific items*. The second focus group resulted in restructuring the scales to replace the *Loss of homeland* section with an *Ambiguous loss* section (see Killikelly et al., 2025S, Appendix 1, Step 2 FG2 outcomes). Framework analysis conducted on the two FGs revealed three major categories with several corresponding subthemes (see Table 2).

**Table 2:** Overview of Framework Analysis Results: Categories and Themes Resulting From FG and CI

Sample / Category	Themes
*Focus groups*	
*Category 1: Item generation and content adaptation*	Content adaptation New content
*Category 2: Clinical utility*	Systematic assessment Treatment priorities
*Category 3: Feasibility*	Duration Cultural differences
*Cognitive interviews*	
*Content validity:* *Category 1: Sources of response error*	Difficulties with response options Inapplicable items Language and meaning
*Content validity:* *Category 2: Expected distress reactions*	Strong emotions Refusal to respond

#### Category 1: Item Generation and Content Adaptation

Clinicians reviewed the content of the existing IPGDS items and made suggestions for new content and for adaptation.

##### Theme: Content Adaptation

Clinicians made several concrete suggestions for how the existing IPGDS could be adapted to the refugee context. Clinicians suggested including a second step in the assessment process which would include assessing grief for the homeland. Several clinicians identified how refugees may experience grief for the loss of a person but also for the loss of their community and culture (homeland).

FG2 Is it the same feeling "grieving for someone who is deceased" and "grieving for someone who we are just separated from"?

Regarding PGD, grieving while being a refugee (how being a refugee can have an impact on the grieving and vice versa) was a main topic. According to the participants, these two experiences overlap and are hardly separable from each other.

FG1 I find it extremely complicated because refugees have an accumulated grief. And that kind of grief we have, it's connected socially, politically or religiously and normally we learn to suppress that and to deal with life as it is. [of note this clinician has a Syrian background so could speak as a cultural broker].

Other additions include revisions to the wording and structure of the scale. Two examples are given below:

FG1 I have another question: “my grief is worse because”, is it possible to make it the other way? “My grief would better if”?

FG2 Will you integrate multiple losses? Because all these scales and criteria have been developed for people who face one loss. In the refugee context, they've lost brother and mother and friend.

##### Theme: New Content

Clinicians also suggested specific items that should be added to the existing ICD-11 criteria including hopelessness, arousal, rituals, and conflicting symptoms. Fifty-seven new items were ultimately added to the catalogue of possible symptoms. These new items included the loss of homeland and the unique challenges experienced by refugees.

FG1 They are kind of preoccupied and at the same time they avoid all reminders. They can have both at the same time. It seems like paradoxical (…).

FG1 This is a kind of avoidance, you kind of shut, you close it, life is over because someone has died. Sometimes I have the feeling when I'm with this lady as if she resigned, she gives up.

Clinicians also mentioned typical circumstances that refugees are confronted with that should be assessed such as multiple losses, ambiguous losses (missing persons) or the lack of resources in the host country.

FG1 One was ambiguous because the husband just was gone, gone until now no one knows what happened to him, if he's dead or he lives with another family somewhere happily or whatever, no one knows.

#### Category 2: Clinical Utility

Clinicians provided insight into how the assessment measure would be useful in the clinic environment.

##### Theme: Systematic Assessment

Clinicians identified that grief is a common complaint amongst refugees and that a questionnaire to assess it in refugees in a more systematic way is missing. They mentioned how they could use the IPGDS for refugees with their patients, for example:

FG2 It's not easy for many of our patients to differentiate between their feelings. For many, stress (or distress) is the most we can get from them. Anger, guilt, shame and grief, that's too much for many of them, so that makes it more difficult to distinguish.

##### Theme: Treatment Priorities

Clinicians reported the need to have a hierarchy of what’s the most important or acute symptom. Refugees often suffer from many problems such as different types of pre and post migration stressors and various types of symptoms. Clinicians must set treatment priorities in terms of what should be treated first and adjust treatment planning accordingly. For example:

FG2 So that's surely an important issue, what is the most prominent symptom, what is causing the most suffering.

Clinicians saw an added value in using pre-post measures of the IPGDS, as this would help to track treatment progress and to indicate which symptoms of PGD would have been improved after a certain treatment.

FG1 What is the main fact […], is it less sadness or acceptance? What is our success?

#### Category 3: Feasibility

Clinicians provided specific feedback on how feasible and achievable a grief assessment for refugees would be in their clinic environments.

##### Theme: Duration

The most common criticism of the questionnaire was the duration. Many clinicians identified that there were too many items and the process may take too long in the clinic. They also pointed out the fact that as many refugees do not speak the language of their host country, clinicians must work with translators which adds extra time.

FG1 So even a simple BDI questionnaire takes about one hour to be translated, so the shorter the better.

All of the clinicians agreed that the IPGDS should be shortened but they also argued that it should be able to capture important content. Clinicians therefore suggested having two separated scales in the IPGDS for refugees, one for the loss of a person and another one for the loss of a homeland. The different results on those scales could be compared:

FG1 (…) then we could apparently differentiate grief or sadness related to a loss of home country or culture and to tear that apart and grief related to the loss of a loved one. That's not the same thing and almost all refugees of course suffer from grief, so to speak related to the loss of their home country or culture (…).

##### Theme: Cultural Differences

Clinicians identified one of the biggest challenges in working with refugees as differences in belief systems and culture. Indeed, clinicians expressed how each of their patients have their own way of expressing their problems or symptoms regarding their cultures or beliefs that are most of the time different from their own. They mentioned how their patients may fear being misunderstood due to cultural differences, and therefore may fear disclosing symptoms.

FG1 I have one Kurdish patient and he says "I see in Switzerland you grieve for 10 days or two weeks, but I would do it for one year. People are expecting me to move on but I'm not ready at the moment".

Another common problem may be patients’ non-acceptance of the problem and, therefore, a non-adherence to treatment. This can make diagnostic and treatment decisions difficult. In the case of PGD, participants also pointed out the multiple ways in which the disorder can manifest itself and, again, this makes its diagnosis difficult.

The use of a reference point or a cultural mediator to understand the culture of the patient was suggested. Indeed, they often use a mediator or someone from the family to help them understand the problem in the patient’s context. The other strategies to develop a common cultural understanding included: to try to step in the patients’ shoes, to confront the patient with the problem, to use psychoeducation, and to plan a treatment in advance.

### Cognitive Interviewing: Preliminary Content Validity

The results from the six cognitive interviews revealed two main categories with several underlying themes. These results are based on both the ‘think aloud’ and probing methods used interchangeably throughout the interviews.

#### Category 1: Sources of Response Error

##### Theme: Difficulties With Response Options

The response options of the IPGDS include a five-point Likert scale (not at all, rarely, sometimes, often, always). During the interviews, participants rarely used those given options and were more likely to answer with “yes”, “no” or with other alternatives. When participants made an effort to answer in the manner requested, confusions or difficulties often occurred. For example, when asking Participant 3 why he chose a specific option, he answered that he chose randomly and didn’t know if the response was really correct for him.

Interviewer I try to avoid reminders of the deceased or the death as much as possible (like photos or memories).

Participant 1 absolutely

##### Theme: Inapplicable Items

Participants identified items that did not apply to their experience of grief or where not relevant. For example, some questions were asked assuming that participants had not attended the funeral of their loved one. However, in many cases participants had been in the same country as the person who died and could be present at the funeral. In this case the question about inability to attend a funeral or other rituals did not apply. The question about visiting the grave of the deceased person was also deemed inapplicable by all participants. For example:

Interviewer I would do anything to feel close to the deceased (e.g., visit their grave every day, sleep next to their picture).

Participant 5 It depends it's too far away, you can't imagine it's not realistic, because the grave it's too far away, I can't go to Syria.

Another example is the question on acceptance:

Interviewer I have trouble or just don’t want to accept the loss.

Participant 1 I don’t really understand the question […] because what does it mean or how should you accept a loss at all? […] Yes, but the second one doesn’t make sense for me. What does it mean to accept it. Like to accept, it means that I’m okay with it? You can’t ask this question. I don’t know, this question is a bit confusing.

The timeframe for assessment was also questioned. Participants were asked to think about the previous week while answering the questions. Most participants had difficulties answering the questions while relating only to the last 7 days.

##### Theme: Language and Meaning

Participants also provided feedback on the language translation. For example:

Interviewer I have intense feelings of sorrow, related to missing my family and friends.

Participant 3 It's more an Egyptian word, not high Arabic. [wrote a different word]

At times participants pointed out when they encountered issues with the wording and how this specific way of writing in Arabic prevented them from answering the question adequately. For example:

Interviewer I have trouble or just don’t want to accept the loss.

Participant 1 There is a difference between “I have trouble or suffer from something” and “I can accept something”.

Some word problems affected all participants whereas others were only mentioned by a single person. In this case, it didn’t require a change after the revision of the questionnaire.

#### Category 2: Expected Distress Reactions Triggered During the Interview

Two main themes, strong emotions (blaming and anger) and rejection of the questionnaire item (guilt, non-acceptance of the item) were observed as reactions to certain items. These are expected distress reactions as we expect certain emotional responses are likely to be triggered if these items are clinically valid and important.

##### Theme: Strong Emotions or Distress

During the interviews, all participants experienced strong emotions or distress at some point. Indeed, one participant felt very emotional when filling out the questions about how her loved one died and when she was asked if the death had been expected or not. Over the course of the interview, most of the participants became more comfortable and were able to relax. However, two participants remained very emotional during the interview process. For example:

Interviewer I’m longing or yearning for the deceased.

Participant 6 Sure. She is always on my mind. [Crying]. It is weird to talk about her with strangers.

In most cases, participants could answer the questions without difficulties and without feeling overwhelmed. However, a few items seemed to provoke strong emotions for all participants.

All participants expressed strong blame towards someone or something for the death of their loved one, or for the reason they had to leave their country. Four out of six participants blamed others or the circumstances for the death and all six participants blamed others or the circumstances for the reason they had to leave the country:

Interviewer I blame others on the circumstances for the death (like a higher power).

Participant 1 I blame the higher powers which are directly responsible for the problems, like the regime. Yes.

Anger was also a strongly expressed emotion. Five out of six participants felt very angry about the loss and, again, all of them reported feeling angry over being separated from their family and friends.

Interviewer I’m angry over the death.

Participant 6 I always say: How did you leave me alone here and leave?

##### Theme: Refusal to Respond

It was observed that several items seemed to trigger a rejection or denial response from participants. One participant in particular had difficulties answering certain questions in general, for example:

Interviewer I feel that I lost a part of myself.

Participant 3 I won't answer.

Nevertheless, for most participants only a few items triggered rejection. Questions about guilt triggered ambivalent responses and seemed to bring up discomfort in all participants. Not only did they all report not feeling guilty for the death of their loved one, but some of them were even shocked or surprised by such a question, as if they would not allow themselves to be guilty:

Interviewer I feel guilty about the death or circumstances surrounding the death.

Participant 5 How should I be guilty of it? No for sure, I'm not guilty. I didn’t have to do anything with the death.

When asked about feeling guilty about being separated from their family and friends, participants were more receptive. Participants acknowledged feelings of guilt.

Importantly, one participant expressed a wish to die. Some items seemed to cause him such distress that he refused to answer. He seemed particularly nervous during the whole interview but items specifically about death or his role in life triggered even more emotions and at times he did not want to answer:

Interviewer I want to die in order to be with the deceased.

Participant 3 I don't want to answer.

#### Change Analysis

In addition to the qualitative framework analysis, we examined the percentage of change in the questionnaire items, afforded by each step of the adaptation process. Here the results show that the number of changes at each step was reduced (e.g. from 80% of items changed in Step 1 to 12.5% changed in Step 3). This supports the content validity of the final questionnaire assessed by Arabic-speaking refugees in Step 3 (cognitive interviews). At this final stage only small changes to translation and some clarifications of content were required.

## Discussion

The IPGDS is the only scale that is extended to include culturally specific symptoms of grief alongside items for a prolonged grief diagnosis (Killikelly & Maercker, 2023). So far, there is no questionnaire to assess PGD and grief more generally, taking the refugee experience into account. This formative, proof of concept, research project had two overarching aims 1) to use a step-by-step method of cultural augmentation to develop an IPGDS addendum tailored to Arabic-speaking refugees and displaced persons (IPGDS-ARD) and 2) To provide an in-depth description of the rationale supporting the new content and additional scales in terms of feasibility, clinical utility, and content validity. To this end we conducted two focus groups with experts and six cognitive interviews with bereaved Arabic-speaking refugees. This resulted in a newly culturally relevant IPGDS-ARD questionnaires for bereaved Arabic-speaking refugees. Of note the current study aimed to develop and extend items for bereaved refugees more generally, however the resulting scales have only been piloted in a small group of Arabic speaking refugees. This will be explored in follow up studies.

**Figure 2:**
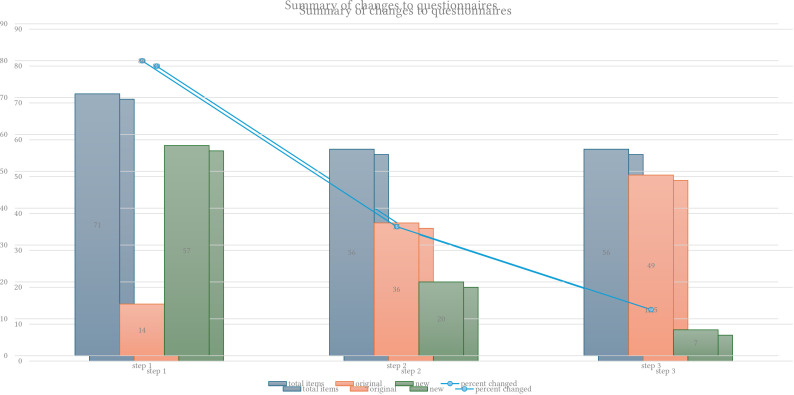
Percentage of Change in Number of Questionnaire Items After Each Augmentation Step

In the first step of cultural augmentation, FG 1, the original IPGDS standard scale measuring ICD-11 PGD symptoms was preserved however the cultural supplement questions were replaced with *a) loss of homeland scale, b) refugee adjustment and impact on grief and c) new culturally specific items*. The rationale for the inclusion of these new sub-scales is captured by the themes revealed in the framework analysis: improved clinical utility (systematic assessment, definition of treatment priorities) and feasibility (duration, cultural differences) for use with bereaved refugees. In the second step, FG 2, the sub-scales were adapted again based on clinical utility and feasibility for use in the busy clinic environment. For example, *the loss of homeland* sub-scale was changed to the *ambiguous loss* sub-scale to reflect a more clinically useful phenomenon. In the final step of the adaptation the CI revealed few changes to the content of the questionnaires. Small changes to translation and some re-phrasings of the items were made. Overall, the few changes from the six CI support the content validity of the final IPGDS-ARD questions (see Figure 2).

Culture is a crucial component for better understanding the expression of illness, that is, cultural differences must be understood and considered for an appropriate diagnosis and treatment (Bhugra, 2005; Bhugra & Becker, 1999). In this study clinicians served as cultural brokers to provide a first window of insight into the clinical utility of a grief measure for refugees. The qualitative analysis on the FGs and CI provided rich data supporting each step of cultural augmentation and restructuring. Clinicians’ recommendations and insights reflected main themes of improving the clinical utility, feasibility, and content validity of the questionnaires. These are touchstones of establishing questionnaire acceptability and validity, particularly for different cultural groups. Previous research has used similar methods including focus groups and CI to develop the content of cultural adapted mental health measures. Our previous work has also demonstrated the value of including a wide range of clinician and patient perspectives to establish cultural relevance and clinical utility of the IPGDS. Swiss and Chinese health care workers examined the content of the ICD-11 PGD guidelines and provided in depth qualitative interview data on their perspectives on the clinical utility and global applicability (Stelzer et al., 2020). Important themes were revealed including the role of stigma in preventing help seeking and treatment and the value of including somatic symptoms in the diagnostic guidelines. Interviews with Japanese health care workers confirmed the overall utility of the ICD-11 PGD guidelines but included important possible barriers to clinical assessment such as the role of emotional control and the strong shame attached to seeking grief support (Killikelly et al., 2023). Health care workers can provide vital insight into their own illness beliefs and beliefs about the usefulness of assessments, as well as insight into their patients experiences and illness models.

The outcome of this cultural adaptation is an addendum to the original standard IPGDS scale with three new subscales: *a) ambiguous loss, b) refugee adjustment and impact on grief and c) culturally specific items.* The *ambiguous loss* section will be an extremely valuable measure for refugees and displaced people. Ambiguous loss is increasingly found to be a significant source of mental distress for displaced people (Boss, 2006). It is defined as the loss of a loved one where death is not confirmed (Solheim et al., 2016). Until now there have been no validated scales or systematically developed measures to assess ambiguous loss in refugees. Renner et al. (2021) found that ambiguous loss is associated with higher levels of depression and prolonged grief. We have built on the preliminary findings from the IPGDS-ARD to develop a shorter stand-alone scale, the AL+ (Comtesse et al., 2023).

The section *refugee adjustment and impact on grief* seeks to examine how post migration experiences may hinder the grieving process as an experience specific to refugees and displaced people. Hwang et al. (2008) recommend that cultural and contextual factors are considered in a timely and consistent manner when treatment planning and that this is re-evaluated systematically. This is especially important because many minorities share similar immigration experiences that could be targeted in prevention and treatment programs. Indeed, in a study about the impact of migration on illness experience and help-seeking strategies of patients from Turkey and Bosnia, Gilgen et al. (2005) examined explanatory models to investigate how those patients understood their illness and found that refugees attributed some of their migration experiences as causes of their illness. Kim et al. (2017) found that traumatic events can impact or diminish the ability to grieve. In their study, participants reported that the psychological sequalae following traumatic events such as torture were more significant and impairing and they saw grief as a less significant problem. The IPGDS-ARD provides clinicians and patients with concrete questions that may help unpick the source of distress and guide further assessment and tailor treatment planning towards grief interventions or towards resource building and support for post migration living stress.

It is important to note that these three new scales should be used in addition to the standard IPGDS scale if a diagnosis is sought. Only the standard IPGDS scale based on the ICD-11 PGD items can be used to confirm a diagnosis. The 3 scales in this new addendum can then be used to guide clinicians to assess and explore other grief related areas of possible concern and distress in refugee groups.

### Limitations

There are several limitations in this study. First, the small sample sizes for the focus groups and cognitive interviews indicate that our results should be considered a preliminary examination. A follow up study is underway to examine the psychometric validity of these new subscales in a larger bereaved refugee sample. In addition, the sample did not include a clinical sample of patients with a confirmed diagnosis of PGD. Additional testing is needed to confirm the validity in a clinical sample of bereaved refugees with PGD. Further adaptations may be necessary to ensure the clinical utility of the subscales for different refugee groups. At the moment there are several items in the subscales which increase the administration time. Further reduction and refinement of the items may be required to improve the clinical utility. The analysis of percentage of change after each adaptation step may be biased as the participants in the CI may be hesitant to express criticism to the research team. As a next step it will be vital to further refine the addendum items through patient and clinician debriefing and to examine clinical decision making and rates of PGD diagnosis with and without the support of the addendum (see the method in Lewis-Fernández et al., 2017).

### Conclusions and Future Directions

In this study, clinicians carefully and thoughtfully described the difficulties they sometimes experience providing accurate culturally reliable assessment and treatment for patients from different cultural backgrounds with a particular focus of bereaved Arabic-speaking refugees. They particularly emphasized the difficulty in assessing and treating PGD in refugees as patients often present with a myriad of symptoms and stressors. The IPGDS for refugees, along with the newly developed subscales (IPGDS-ARD) were presented to assess PGD and grief experiences more wholistically in Arabic-speaking refugees and with the aim to support other displaced people in the future. The new IPGDS-ARD will help clinicians diagnose PGD with the standard scale as well as assess the importance and relevance of possible overlapping or co-occurring stressors such as post migration difficulties or ambiguous loss. Due to the challenges with clinical utility including the large number of items, we recommend using a formulation approach to develop a symptom map of the most distressing and clinically relevant symptoms highlighted through both the standard IPGDS scale and the addendum. An example formulation is included in Appendix 2 (see Killikelly et al., 2025S). Additionally, the newly developed and tested Ambiguous Loss Inventory + would directly assess the loss of missing loved ones (Comtesse et al., 2023). Clinicians will then be able to direct treatment to appropriate evidence-based interventions.

## Supplementary Materials

The Supplementary Materials contain the online appendices for the study (see Killikelly et al., 2025S):

- **Appendix 1. Summary of questionnaire changes after each step, resulting in the Addendum for Refugees and Displaced people (IPGDS-ARD):** Here we present the process of item reduction and generation resulting in the final version of the addendum.
- **Appendix 2. Sample formulation template for assessing grief in displaced people:** Here we present a template formulation for considering treatment planning for working with grief and displaced people.

KillikellyC.
ReymondA.
AeschlimannA.
MaerckerA.
HeimE.
 (2025S). Supplementary materials to "International Prolonged Grief Disorder Scale Addendum for Refugees and Displaced people (IPGDS-ARD): A study of Arabic-speaking bereaved refugees"
[Online appendices]. PsychOpen. 10.23668/psycharchives.15956
PMC1196056840177334

## Data Availability

Data are available upon request.

## Reporting Guidelines

Reporting standards for quantitative research in psychology: The APA Publications and Communications Board task force report.

## Preregistration

The study was not preregistered.

## Related Versions

This manuscript is based on the master’s thesis of AR: Reymond, A. (2020). *Development and preliminary testing of the International Prolonged Grief Disorder Scale for refugees* [Unpublished master’s thesis, University of Zurich, Department of Psychology].
